# NF-kappaB activation is associated with homocysteine-induced injury in Neuro2a cells

**DOI:** 10.1186/1471-2202-9-62

**Published:** 2008-07-07

**Authors:** Nadia Ferlazzo, Salvatore Condello, Monica Currò, Giulia Parisi, Riccardo Ientile, Daniela Caccamo

**Affiliations:** 1 Department of Biochemical, Physiological and Nutritional Sciences, University of Messina – Messina, Italy

## Abstract

**Background:**

Perinatal exposure to hyperhomocysteinemia might disturb neurogenesis during brain development and growth. Also, high levels of homocysteine trigger neurodegeneration in several experimental models. However, the putative mechanisms of homocysteine-induced toxicity in the developing nervous system have poorly been elucidated. This study was aimed to investigate homocysteine effects in undifferentiated neuroblastoma cells, Neuro2a.

**Results:**

A 4 h exposure to homocysteine in a concentration range of 10–100 μM did not affect cell viability and ROS production in Neuro2a cell cultures. Instead, ROS levels were increased by two-three folds in cells treated with 250 μM and 500 μM homocysteine, respectively, in comparison with control cells. Also, the highest homocysteine dose significantly reduced the viable cell number by 40%. Notably, the treatment with homocysteine (250 μM–500 μM) in the presence of antioxidants, such as N-acetylcysteine and IRFI 016, a synthetic α-tocopherol analogue, recovered cell viability and significantly reduced homocysteine-evoked increases in ROS production. Moreover, antioxidants, particularly IRFI 016, were able to counteract NF-κB activation induced by 250 μM homocysteine.

Cell treatment with 250 μM homocysteine also triggered the onset of apoptosis, as demonstrated by the increased expression of early apoptotic markers such as Bax, caspase-3 and p53. In contrast, Bcl2 expression was not affected by homocysteine exposure. Interestingly, the specific inhibition of NF-κB nuclear translocation by the synthetic peptide SN50 was able to almost completely suppress the homocysteine-evoked rises in pro-apoptotic protein expression as well as in caspase-3 activity. Further, also IRFI 016 and N-acetylcysteine were able to significantly reduce caspase-3 activation induced by homocysteine treatment.

**Conclusion:**

These observations suggest an involvement of redox state alterations and activated NF-κB in apoptosis onset triggered by homocysteine in neuroblastoma cells Neuro2a. However, further investigations are needed to characterize molecular events involved in the NF-κB activation induced by homocysteine.

## Background

Homocysteine (Hcy) is a non-proteic sulfur-containing amino acid product of methionine metabolism. Hyperhomocysteinemia is determined by genetic factors, such as deficiency in enzyme activities involved in homocysteine remethylation and transulphuration pathways, and/or reduced dietary intake of folate, vitamin B6 and vitamin B12 [1]. Increased plasma Hcy levels are a well known risk factor for cardiovascular diseases, and have also been associated with neurodegenerative disorders, such as Alzheimer's disease and Parkinson's disease, and cognitive dysfunction in the elderly [2-4]. Moreover, perinatal exposure to hyperhomocysteinemia might disturb neurogenesis during brain development and growth, as well as hereditary severe hyperhomocysteinemia leads to a variety of neurological impairments in children [5,6]. However, the putative mechanisms of homocysteine-induced toxicity in the developing nervous system have poorly been elucidated.

Hcy induces neurotoxicity in human and murine neuronal cells through several mechanisms, including over-stimulation of NMDA receptors, oxidative stress and DNA damage [7-9]. Hcy is excitotoxic at least as glutamate and also enhances glutamate excitotoxicity [9]. Moreover, while normal activation of NMDA receptors can provoke both excitation and inhibition, Hcy only elicited excitation in cerebellar neurons [7].

Recently, a novel mechanism for Hcy neurotoxicity has recently been described. This involves the inhibition of cytochrome oxidase activity following copper binding by Hcy [10]. Also, previous studies demonstrated that high Hcy levels cause an imbalance in the redox status homeostasis with generation of toxic reactive oxygen species (ROS) [11]. The excess of ROS production triggers cell cycle arrest and apoptosis, finally leading to neurodegeneration [12].

A growing body of evidence suggests that ROS-induced cell damage results from or is accompanied by inflammatory processes. It has been shown that NF-κB activation plays a pivotal role in the up-regulation of many inflammatory genes, whose products are putatively involved in apoptosis [13]. However, the role of NF-κB in neuronal death is controversial, and the effects of NF-κB nuclear translocation in cell response still remain unclear. Indeed, it has been reported that NF-κB activation in neurons is protective against degeneration, whereas in microglia promotes cell death [13].

The present study was designed to investigate the role of pro-oxidant effects of Hcy exposure in undifferentiated neuroblastoma cells, Neuro2a, and the possible involvement of NF-κB pathway in Hcy-induced cell injury.

## Methods

### Materials

Eagle's Minimal Essential Medium (MEM), foetal bovine serum (FBS) and antibiotics were from Invitrogen Life Technologies (Milano, Italy). The caspase-3 substrate Ac-Ala-Ala-Val-Ala-Leu-Leu-Pro-Ala-Val-Leu-Leu-Ala-Leu-Leu-Ala-Pro-Asp-Glu-Val-Asp-p-nitroaniline (DEVD-pNa), D, L-Hcy, N-acetylcysteine (NAC), non-enzymatic cell dissociation solution, and other chemicals of analytical grade were from Sigma (Milano, Italy). Rabbit polyclonal antibody against Bax was from Chemicon (USA). Mouse monoclonal antibodies against Bcl-2 and caspase-3 were from BioVision (Mountain View, USA). Rabbit polyclonal antibody against p53, mouse monoclonal antibodies against β-actin as well as p50 (sc-8414X) and p65 (sc-8008X) NF-κB subunits, and the NF-κB inhibitor, SN50, were from Santa Cruz Biotechnology (DBA, Milano, Italy). The oligonucleotide probe containing the NF-κB consensus sequence present in the κ-light chain promoter was synthesised by MWG Biotech (Monza, Italy). Kodak X-ray film was from Kodak (Milano, Italy). IRFI 016, a synthetic α-tocopherol analogue, was generously supplied by Biomedica Foscama Research Centre (Frosinone, Italy).

### Cell culture and treatment

Mouse neuroblastoma cells Neuro2a (ATCC-CCL 131) were grown in MEM, supplemented with 10% (vol/vol) FBS, 2 mM L-glutamine, 1 mM sodium pyruvate, 50 μg/ml streptomycin and 50 U/ml penicillin, at 37°C in a 5% CO_2_/95% air humidified atmosphere. Cell cultures were maintained for at least two weeks, with medium renewal every two days.

Subconfluent cells were treated with Hcy (10–500 μM) for 4 h in the presence or absence of antioxidant compounds, such as NAC (500 μM) and IRFI 016 (80 μM), which were added to the culture medium 30 min prior to Hcy treatment. Four replicates were performed for each sample.

A subset of experiments was carried out using SN50 (50 μg/ml), a synthetic peptide able to specifically inhibit the nuclear translocation of NF-κB, that was added to cell cultures 30 min prior to Hcy exposure.

### ROS production

In order to evaluate ROS production, Hcy-treated or untreated Neuro2a cells, cultured in 12-well culture plates, were incubated for 30 min with dichlorodihydrofluorescein diacetate (H_2_DCF-DA) (5 μM). Then, cells were washed with phosphate-buffered saline (PBS), detached with non-enzymatic cell dissociation solution, and resuspended in 300 μl of PBS supplemented with 0.1 M KH_2_P0_4 _and 0.5% Triton X-100. Supernatants were recovered by centrifugation at 13,000 rpm for 10 min, and analyzed under fluorescein optics, as previously described [14].

### Cell viability assay

To assess Hcy effects on cell viability, an MTT (3-(4, 5-methylthiazol-2-yl)-2, 5-diphenyl-tetrazolium bromide) reduction assay was performed. After Hcy treatment, Neuro2a cells, grown in 96-well culture plates, were incubated with fresh medium containing MTT (0.5 mg/ml) at 37°C for 4 h. Then, insoluble formazan crystals were dissolved in 100 μl of a 10% (w/v) SDS solution in HCl 0.01 M. The optical density in each well was evaluated by spectrophotometrical measurement at 540 nm with a Sunrise microplate reader (Tecan Italia, Cologno Monzese, Italy). The percent cell viability was assessed by the absorbance ratio of Hcy-treated vs untreated cells. All experiments were made in triplicate.

### Western blotting

After treatment, Neuro2a cells were homogenized on ice. Proteins (30 μg) were separated by SDS-PAGE onto 8.5% gel, and transferred to nitrocellulose membranes. Blots were blocked overnight at 4°C with 5% non-fat dry milk; then, membranes were incubated for 1 h at room temperature, on a rotating device, with mouse monoclonal antibodies against either Bcl-2, or caspase-3 or β-actin (diluted 1:1000 in TBS-T) and rabbit polyclonal antibodies against either Bax or p53 (diluited 1:2000 and 1:200, respectively, in TBS-T), followed by incubation for 1 h with horseradish peroxidase-conjugated anti-mouse IgG (1:1500 for Bcl-2, and 1:2500 for caspase-3 and β-actin, in TBS-T) or anti-rabbit IgG (1:2500 for Bax, and 1:1000 for p53, in TBS-T). Blots were developed by ECL chemiluminescence detection system kit using Kodak film. Bands were scanned and quantified by densitometric analysis with an AlphaImager 1200 System (Alpha Innotech, San Leandro, CA, USA), after normalisation against β-actin.

### Caspase-3 activity assay

After cell lysis, insoluble proteins were removed by centrifugation at 1,000 × g for 3 min at 4°C. A 50 μl aliquot of 2× reaction buffer/DTT mix, and 5 μl of 1 mM caspase-3 substrate DEVD-pNa (final concentration 50 μM) were added to the supernatants. Samples were incubated at 37°C for 60 min, and then DEVD-pNa cleavage was evaluated by measuring sample absorbance at 405 nm, using DU 800 spectrophotometer (Beckman Coulter, Fullerton, CA, USA). Measurements were calibrated against a standard linear regression curve of p-Na. Caspase activity was defined as nmol pNA released per hour per mg of protein (nmol/h/mg protein).

### Electrophoretic mobility shift assay (EMSA)

The presence of NF-κB DNA binding activity in cell nuclear extracts was evaluated by EMSA and supershift assay, as described by Caccamo et al. [15].

### Statistical analysis

All values are presented as means ± SEM. Statistical analysis was performed using one-way ANOVA, followed by Newman-Keuls post-hoc test.

## Results

**Figure 1:**
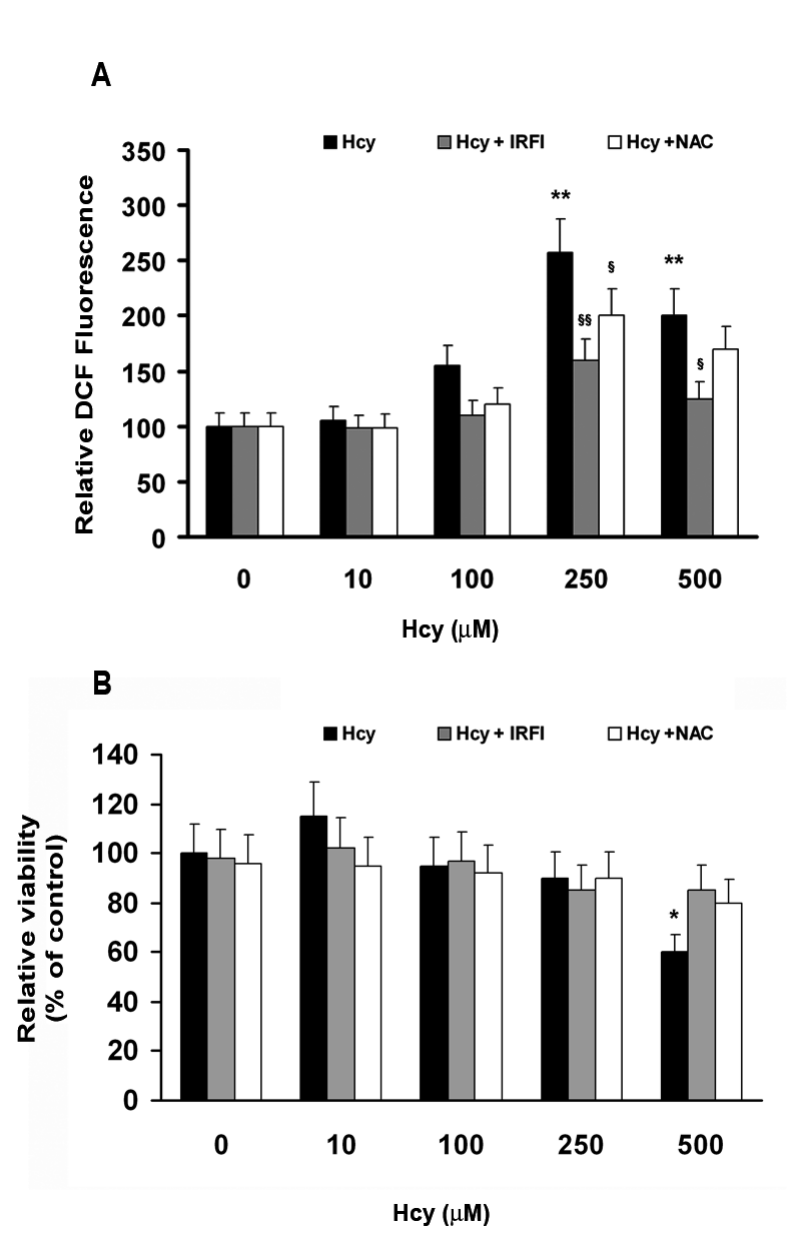
**Effects of Hcy on ROS production (A) and cell viability (B) in neuroblastoma Neuro2a cells**. ROS production was measured by DCF formation as reported by Lee et al. [14]. Cell viability was evaluated by MTT assay as described in Materials and Methods. Results shown are mean ± SEM of three-five separate experiments. *p < 0.05 and **p < 0.01 significant differences in comparison with untreated cells; ^§^p < 0.05 and ^§§^p < 0.01 significant differences in comparison with Hcy-treated cells.

To evaluate Hcy-induced cell redox status alterations, Neuro2a cell cultures were exposed to different Hcy doses (10–500 μM), in the presence or absence of NAC (500 μM) and IRFI 016 (80 μM). Hcy did not significantly affect ROS levels in Neuro2a cells when used in a concentration range of 10–100 μM. Higher Hcy concentrations, i.e. 250–500 μM, produced a two-three fold increase in ROS production, respectively, in comparison to untreated cells. The incubation of cell cultures with Hcy in the presence of either NAC or IRFI 016 was able to strongly counteract Hcy effects on ROS production (Fig. 1A). Notably, a higher protective effect was displayed by IRFI 016, reducing Hcy-evoked increases in ROS levels by 35%, compared with a 20% reduction achieved in the presence of NAC (Fig. 1A).

The MTT assay demonstrated that the treatment with 10–250 μM Hcy did not significantly affect the number of viable cells compared with controls, while 500 μM Hcy drastically reduced cell viability to less than 40% (Fig. 1B). Notably, IRFI 016 and NAC were able to recover cell viability (Fig. 1B). The subsequent experiments were carried out using 250 μM Hcy, in order to minimize alteration in cell homeostasis dependent on massive cell necrosis.

**Figure 2:**
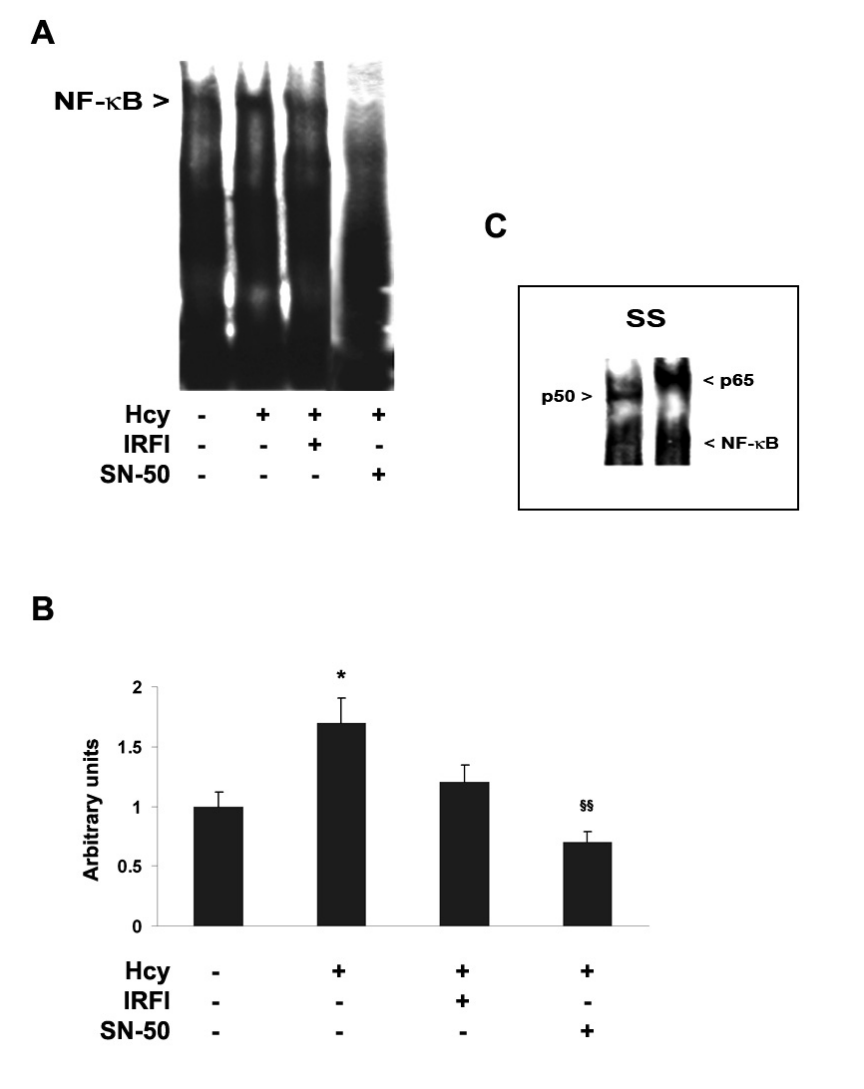
**Hcy treatment elicited NF-κB activation in Neuro2a cells**. A) **Detection by EMSA of NF-κB DNA binding activity in nuclear extracts of untreated or Hcy-treated Neuro2a cells**. After a 4 h incubation with 250 μM Hcy, in the presence or absence of either IRFI 016 (80 μM) or the NF-κB specific inhibitor SN50 (50 μg/ml), NF-κB activation was evaluated by incubation of nuclear proteins (6 μg) with a 40-mer biotinylated oligonucleotide probe containing the NF-κB consensus sequence. B) Densitometric analysis of NF-κB activated complexes. The amount of NF-κB activated complexes was significantly increased by Hcy treatment. Notably, IRFI 016 as well as SN50 strongly reduced Hcy-increased NF-κB levels. Results shown were from three separate experiments. *p < 0.05 significant differences in comparison with untreated cells, and ^§§^p < 0.01 significant differences in comparison with Hcy-treated cells. C) Supershift analysis of NF-κB activated complexes. A supershift assay, carried out by incubating nuclear proteins with specific monoclonal antibodies against p50 and p65 NF-κB subunits, demonstrated that NF-κB activated complexes were p50/p65 heterodimers.

The possible occurrence of NF-κB activation, concomitantly with redox state alterations induced by Hcy, was investigated by EMSA. We demonstrated an increase in DNA binding activity by heterodimer NF-κB complexes, mainly composed by p50 and p65 subunits, in nuclear extracts of Hcy-treated neuroblastoma cells in comparison with untreated ones, where lower amounts of active NF-κB complexes were found (Fig. 2A–C). Moreover, as expected, the incubation with SN50 significantly inhibited NF-κB nuclear translocation (Fig. 2A–B). Notably, when Neuro2a cells were incubated with Hcy (250 μM) in the presence of IRFI 016 (80 μM), a significant reduction of NF-κB levels was observed (Fig. 2A–B). These data suggest a direct relationship between Hcy-induced oxidative stress and NF-κB activation.

**Figure 3:**
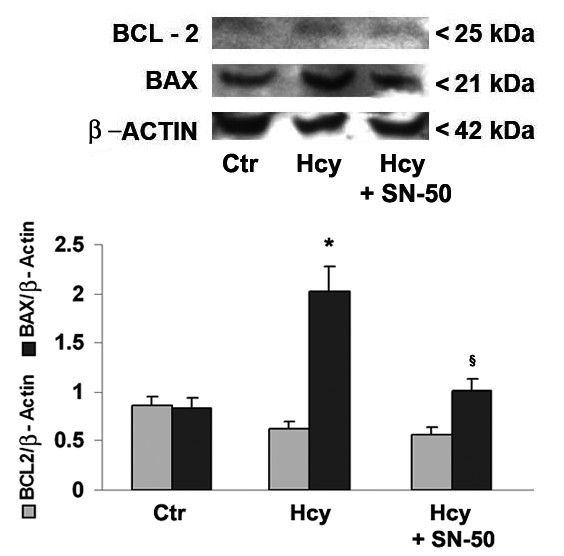
**Effects of Hcy treatment on Bcl-2 and Bax expression in Neuro2a cells**. Neuro2a cells were treated with 250 μm Hcy for 4 h in the presence or absence of SN50, the specific NF-κB inhibitor, that was added to cell cultures 20 min prior to incubation with Hcy. The immunoblots, with relative densitometric analysis after normalization against β-actin, show Hcy-evoked effects on the expression of anti-apoptotic and pro-apoptotic markers, such as Bcl-2 and Bax, respectively. Results are expressed as mean ± SEM of five independent experiments. *p < 0.05 compared with control cells, ^§^p < 0.05 in comparison with Hcy-treated cells.

In order to better evaluate Hcy effects on cell damage in Neuro2a cells, we examined the expression of known protein markers of apoptosis onset. The treatment with Hcy (250 μM) for 4 h significantly increased Bax protein levels in comparison with control cells, whereas Bcl-2 expression was not affected (Fig. 3). Interestingly, the incubation with Hcy in the presence of SN50 reduced Hcy-induced Bax up-regulation while did not affect Bcl-2 levels (Fig. 3).

**Figure 4:**
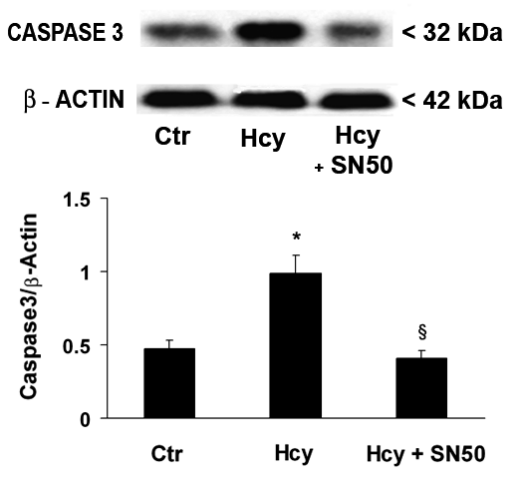
**Western blot analysis of caspase-3 expression in Hcy-treated and untreated Neuro2a cells**. After a 4 h Hcy treatment, in the presence or absence of the specific NF-κB inhibitor SN50, cell proteins were probed with monoclonal antibodies against caspase-3 and β-actin (*figure top*). A densitometric analysis of immunoblots (*figure bottom*) was also carried out after normalization against β-actin. Results were similar in three separate experiments. *p < 0.05 significant difference in comparison with untreated cells; ^§^p < 0.05 in comparison to Hcy-treated cells.

The treatment of Neuro2a cells with Hcy also caused the caspase-3 up-regulation (Fig. 4), that was accompanied by a significant increase in caspase-3 activity compared with control cells (Table 1).

**Table 1:** Caspase-3 activity in Neuro2a cells exposed to Hcy.

	pNa amounts (nmol/h/mg prot)
Control	60 ± 3.3
+ Hcy (250 μM)	142 ± 15.1 **
+ Hcy (250 μM) + SN50 (30 μg/ml)	87 ± 9.5 *
+ Hcy (250 μM) + IRFI 016 (80 μM)	96 ± 7.9 *
+ Hcy (250 μM) + NAC (500 μM)	102 ± 9.2 *
SN50 (30 μg/ml)	53 ± 2.9
IRFI 016 (80 μM)	42 ± 3.4

The evaluation of caspase-3 activity was carried out based on measurements of specific cleavage of DEVD-pNa as described in Materials and methods. Results are mean ± SEM of six experiments. * p < 0.05 and **p < 0.01 significant differences in comparison to control cells.

Interestingly, in Hcy-exposed cells, the SN50-mediated specific NF-κB inhibition lowered high levels of caspase-3 protein (Fig. 4) and enzyme activity (Table 1). The addition of either IRFI 016 (80 μM) or NAC (500 μM) was also able to reduce Hcy-induced increases in caspase-3 activity by 32% and 28% respectively (Table 1).

**Figure 5:**
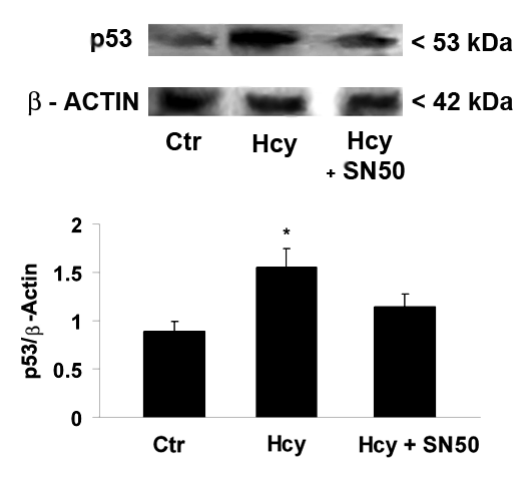
**Western blot analysis of p53 expression in Hcy-treated and untreated Neuro2a cells**. After a 4 h Hcy treatment, in the presence or absence of SN50, the specific NF-κB inhibitor, cell proteins were probed with monoclonal antibodies against p53 and β-actin (*figure top*). A densitometric analysis of immunoblots (*figure bottom*) was also carried out after normalization against β-actin. Results were similar in three separate experiments. *p < 0.05 significant difference in comparison with untreated cells.

To further evaluate the NF-κB involvement in Hcy-evoked apoptosis in Neuro2a cells, Hcy effects on the expression of p53 were assessed in the presence or absence of SN50. Indeed, Hcy treatment induced p53 up-regulation, that was strongly reduced in the presence of SN50 (Fig. 5).

## Discussion

In this study, we demonstrated that exposure to micromolar Hcy concentrations triggered redox state alterations in Neuro2a cell cultures. One cannot exclude that the use here of D, L-homocysteine might have led to somewhat unphysiological results. However, D, L-homocysteine has been widely used in several experimental models, and the results observed are superimposable with physiological responses. Indeed, our observations agree with previous results showing a ROS-mediated alterations following Hcy exposure [16]. Although the precise mechanisms by which Hcy increases oxidative stress are not yet completely understood, the prevention of Hcy-induced toxicity by catalase suggests that hydrogen peroxide plays a role as a mediator of oxidative injury leading to apoptosis [17-19]. Indeed, folic acid supplementation has a protective effect on Hcy-induced oxidative stress by reducing intracellular superoxide levels and, to a lesser extent, quenching hydrogen peroxide [20]. Moreover, Hcy, inhibiting the expression of antioxidant enzymes as well as the synthesis of radical scavengers (e.g. glutathione peroxidase, superoxide dismutase), might potentiate the toxic effects of ROS [21]. Also, Hcy decreases the tissue levels of vitamins A, C and E, thereby reducing the antioxidant reserves [22].

On the other hand, antioxidant treatment with NAC and vitamin E can prevent oxyradical generation in neuroblastoma cells related to increased Hcy or reduced folic acid [22]. Based on these evidence, we chose to test Hcy effects in the presence of the antioxidant IRFI 016, a synthetic vitamin E analogue, that has previously been demonstrated to be protective against oxidative stress induced by excitotoxic amino acid [23]. Interestingly, IRFI 016 was more effective than NAC, the precursor of intracellular glutathione, in reducing Hcy-increased ROS levels. Further, IRFI 016 was able to reduce NF-κB nuclear translocation. These effects suggest a cross-talk between oxidative stress and inflammatory cascade in the alterations triggered by Hcy in Neuro2a cells.

In human hepatoma cells, it has been reported that folate deficiency-induced apoptosis is proceeded via the enhanced activation of NF-κB, which results from the Hcy-mediated overproduction of hydrogen peroxide [24]. In this study, we also demonstrated that the oxidative stress induced by Hcy in Neuro2a cells involves the activation of NF-κB, which is inhibited by the antioxidant IRFI 016. Indeed, NF-κB is a redox-sensitive transcription factor which is crucial in the control of ROS-mediated apoptosis. Previous reports have shown that NF-κB activation is a part of recovery processes after acute oxidative stress induced by various stimuli, other than excitoxicity, in astroglial cells [15,25-29]. Interestingly, depending on the conditions and the cell type considered, NF-κB activation has been shown to have opposite effects on the brain cell survival. In fact, it has been shown that NF-κB inhibition leads to loss of neuroprotection as well as activated NF-κB induces the expression of anti-apoptotic Bcl-2 gene [30,31]. On the contrary, the activation of NF-κB and increase in the mRNA levels of pro-apoptotic Bax have also been demonstrated in different stress conditions including amyloid beta (Aβ)-induced oxidative stress in microglial cells [32].

Among the κB-responsive genes possibly involved in the control of neuronal cell death, various pro-apoptotic genes are activated upon oxidative stress, and might mediate NF-κB/Rel-induced cell death. Here, we observed that the Hcy-induced NF-κB activation mediated the increase in early protein markers of apoptosis onset, such as Bax, caspase-3 and p53.

The cysteine proteases, caspases, are considered to play a central role in the apoptotic processes, and trigger the apoptotic cascade of proteolytic cleavage events in mammalian cells [33]. The most widely studied member of the caspase family, caspase-3, is one of the key apoptosis executioners that is responsible for the partial or total proteolytic cleavage of many proteins. In addition, the p53 protein plays an important role in genotoxic stress, growth arrest, and cell death. Translocated NF-κB may affect p53 phosphorylation, and subsequently, the decrease of NF-κB activation lead to reduced p53 activity, which had been confirmed on neurons exposure to DNA damage [35].

## Conclusion

NF-κB activation seems to be a critical event in the onset of Hcy-induced apoptosis in neuroblastoma cells Neuro2a, given the significant decrease of apoptotic marker expression observed in the presence of SN50-mediated specific NF-κB inhibition. Interestingly, the protective effect displayed by the antioxidants IRFI 016 and NAC, reducing caspase-3 activity, underscore a major role for oxidative stress in Hcy toxic effects.

However, the increase in NF-κB DNA binding activity in apoptotic cells did not address cause-effect relationships. Considering the dual role of NF-κB in cells survival further investigations are needed to elucidate its function in Hcy-induced neuronal cell death.

## Authors' contributions

NF and SC contributed equally to this work. DC designed the study together with RI, participated in its coordination, and drafted the manuscript, with substantial contributions of NF. SC also followed cell culture experiments. MC and GP performed mRNA isolation, RT-PCR experiments and evaluation of ROS production. SC carried out EMSA and immunoblotting experiments, supervised by NF, who also made densitometric analysis. RI and DC were the coordinators of the study, and gave the final approval of the version to be published. All authors read and approved the final manuscript.
